# Motivational Interviewing with computer assistance as an intervention to empower women to make contraceptive choices while incarcerated: study protocol for randomized controlled trial

**DOI:** 10.1186/1745-6215-13-101

**Published:** 2012-07-02

**Authors:** Jennifer Clarke, Melanie A Gold, Rachel E Simon, Mary B Roberts, LAR Stein

**Affiliations:** 1 Brown University Center for Primary Care and Prevention, Pawtucket, RI, USA; 2 Memorial Hospital of Rhode Island, Pawtucket, RI, USA; 3 Department of Pediatrics, Division of Adolescent Medicine, University of Pittsburgh School of Medicine, Staff Physician, Division of Student Affairs, Pittsburgh, PA, USA; 4 Department of Psychology, University of Rhode Island, Kingston, RI, USA

## Abstract

**Background:**

Unplanned pregnancies and sexually transmitted infections (STIs) are important and costly public health problems in the United States resulting from unprotected sexual intercourse. Risk factors for unplanned pregnancies and STIs (poverty, low educational attainment, homelessness, substance abuse, lack of health insurance, history of an abusive environment, and practice of commercial sex work) are especially high among women with a history of incarceration. Project CARE (Contraceptive Awareness and Reproductive Education) is designed to evaluate an innovative intervention, Motivational Interviewing with Computer Assistance (MICA), aimed at enhancing contraceptive initiation and maintenance among incarcerated women who do not want a pregnancy within the next year and who are anticipated to be released back to the community. This study aims to: (1) increase the initiation of highly effective contraceptives while incarcerated; (2) increase the continuation of highly effective contraceptive use at 3, 6, 9, and 12 months after release; and (3) decrease unsafe sexual activity.

**Methods/Design:**

This randomized controlled trial will recruit 400 women from the Rhode Island Department of Corrections (RI DOC) women’s jail at risk for an unplanned pregnancy (that is, sexually active with men and not planning/wanting to become pregnant in the next year). They will be randomized to two interventions: a control group who receive two educational videos (on contraception, STIs, and pre-conception counseling) or a treatment group who receive two sessions of personalized MICA. MICA is based on the principles of the Transtheoretical Model (TTM) and on Motivational Interviewing (MI), an empirically supported counseling technique designed to enhance readiness to change targeted behaviors. Women will be followed at 3, 6, 9, and 12 months post release and assessed for STIs, pregnancy, and reported condom use.

**Discussion:**

Results from this study are expected to enhance our understanding of the efficacy of MICA to enhance contraceptive initiation and maintenance and reduce sexual risk-taking behaviors among incarcerated women who have re-entered the community.

**Trial registration:**

NCT01132950

## Background

Almost half of all pregnancies in the United States are unplanned, a higher percentage than in several other industrialized countries [1,2]. Seventy percent of women between 18 and 29 years of age are at risk of unplanned pregnancy [3], a higher proportion than any other age group [4]. The social and financial costs of unplanned pregnancies are staggering. In 2006, 64% of births in the Unites States resulting from unintended pregnancies were publicly funded, compared with 48% of all births and 35% of births resulting from intended pregnancies. Of the 2 million publicly funded births, 51% resulted from unintended pregnancies, accounting for $11.1 billion in costs [5]. Unplanned pregnancies have been associated with negative antenatal behaviors and birth outcomes [6-10].

Sexually transmitted infections (STIs) are some of the most commonly reported diseases in the United States with approximately 19 million cases occurring annually [11]. STIs can cause serious and even life-threatening sequelae including cancer, ectopic pregnancy, infertility, chronic pelvic pain, spontaneous abortion, stillbirth, low birth weight, prematurity, congenital and perinatal infections, neurological damage, and death. More recent data indicate that many STIs increase the risk of HIV transmission at least three- to five-fold [12]. The economic cost of STIs is staggering. Women, minority populations, and adolescents are disproportionately affected by STIs. The incidence of STIs and their sequelae are consistently higher among African Americans and Hispanic Americans than among Caucasians [11]. In addition, two-thirds of all STIs occur in people younger than 25 years of age [13].

Previous research indicates that both unplanned pregnancies and STIs disproportionately affect poor and medically underserved women, especially the incarcerated population [14]. Among incarcerated women, risk factors for unplanned pregnancies are very high (low educational attainment, poverty, homelessness, and substance abuse) [15] and over 80% of incarcerated women at risk for an unplanned pregnancy report a history of an unplanned pregnancy. Many women with incarceration histories lose custody of their children either because of substance abuse, lack of resources, or re-incarceration. Approximately half of women in prison and jail are between the ages of 18 and 34 years. Over three-quarters of women in jail at risk for an unplanned pregnancy report they want to start a contraceptive method [16]. Furthermore, national statistics of STIs among incarcerated women reveal rates of chlamydia and gonorrhea at 7.2% and 1.6%, respectively [17]. STI rates vary by state but are 12 to 16 times higher in prisons and jails than in the general population [17].

Women comprise approximately 3.2 million arrests annually and many pass through prisons and jails. With so many women at high risk for unplanned pregnancies and STIs passing through United States jails annually, improving contraceptive service utilization and STI prevention services in this non-traditional setting has the potential to reach the women in the greatest need of services. We will conduct a randomized controlled trial (RCT) of an innovative intervention based on motivational interviewing aimed at enhancing contraceptive initiation and maintenance among incarcerated women who do not want a pregnancy within the next year and who are anticipated to be released back to the community.

### Motivational Interviewing (MI) to decrease unplanned pregnancies and STIs

MI provides an empirically supported style for matching counseling to an individual’s readiness to change. Based on the principles of motivational psychology, client-centered therapy, and the Transtheoretical Model (TTM), [18,19] MI represents a general and practical approach for facilitating behavior change by enhancing and eliciting a client’s own internal motivation for change. [20] Responsibility for changing a behavior is assumed to lie within the individual, and ambivalence is recognized as a natural part of this change process. MI is designed to assist clients in working through ambivalence and in moving toward change. Their ambivalence may reflect a desire for a pregnancy in which case the MI counselor changes focus to having a healthy pregnancy. Utilizing MI techniques, the counselor helps the participant identify barriers to using birth control and methods for overcoming those barriers. MI targets increasing interest and confidence in accessing and using condoms and other forms of birth control and use of family planning professionals. Elements of the protocol are tailored to a woman’s specific needs including holding off on pregnancy until she is ready (for example, is financially stable or has a steady partner); it focuses on good and not-so-good ways regarding how to effectively negotiate use of contraceptives with partners (not so good would be to carry a knife to dissuade aggressive partners), decreasing use of alcohol and other drugs during sex (which may make it harder to use birth control and condoms), increasing use of referrals to family planning clinics, and identifying barriers specific to women and generating possible solutions. The MI counselor utilizes techniques including providing personalized feedback, reflective listening, exploring pros and cons of change, giving affirmations, supporting client autonomy and self-efficacy, eliciting ‘self-motivational statements’ (problem recognition, intention to change, optimism about change), and generating solutions to potential barriers to change. Of critical importance, MI emphasizes client’s personal choice regarding change, de-emphasizes labeling the client and her behaviors, and avoids arguing with or confronting the client with the need to change.

Published studies include successful MI interventions with individuals who smoke tobacco [21], those who are addicted to heroin on methadone [22], psychiatric inpatients with and without co-morbid substance use disorders [23], and obese women with non-insulin dependent diabetes [24]. MI is especially effective with individuals who are ‘resistant’ [25], ‘angry’ [26], or not ready to change because the therapeutic process includes recognition and resolution of ambivalence about change. Brief interventions using MI have been postulated to be particularly well-suited to incarcerated populations [27], because of their brief duration, non-confrontational, and empathic therapist style, emphasis on facilitating the individual’s consideration of the effects of their behavior on other life areas, and allowance for multiple options for change.

There are currently few published studies evaluating the use of brief interventions to alter overall contraceptive behavior. Belcher and colleagues evaluated the effectiveness of a motivational skill-based intervention to decrease sexual risk behavior in adult women as compared to a standard educational intervention [28]. Women randomized to MI reported significantly higher rates of condom use and had significantly fewer reports of unprotected intercourse as compared to controls. Reported condom use for women in the MI group rose from 22% to 66% as compared to an increase of 27% to only 43% among controls at 3 months post-intervention (*P* <0.02).

A Centers for Disease Control study entitled ‘Project CHOICES’ evaluated the impact and acceptability of a four-session MI intervention (*n* = 190) designed to reduce alcohol-exposed pregnancies among high-risk women by targeting both contraception use and drinking [29]. Among women who completed the 6-month follow-up, 68.5% were no longer at risk of having an alcohol-exposed pregnancy; 12.6% reduced drinking alone; 23.1% used effective contraception alone; and 32.9% reported both behaviors.

A total of three RCTs assess the impact of MI on women’s contraceptive behaviors in the context of reducing alcohol-exposed pregnancy (AEP). Ingersoll *et al*. reported their preliminary findings from a RCT of a single-session MI-based intervention to reduce AEP risk among college women (18 to 24 years) [30]. Significantly fewer women in the control group (48%) reported using effective contraception at 1-month follow-up as compared to those in the MI group (64%), *P* <0.03. Significantly more participants in the MI group (74%) were no longer at risk for AEP at 1 month compared to control participants (54%), *P* <0.005. This study demonstrated the potential of MI to alter contraceptive risk-taking behaviors among young women in college.

Peterson *et al*. conducted a RCT of a two-session MI-based intervention to reduce the risk of unintended pregnancy and STIs among women aged 16 to 44 years [31]. No significant differences were found between the intervention and control groups between baseline and 12 months. They concluded that additional MI sessions may be necessary to improve contraceptive decision-making and to reduce the risk of unintended pregnancies and STIs [31]. Barnet *et al*. investigated the effectiveness of a computer-assisted motivational intervention in preventing rapid subsequent birth to adolescent mothers. They randomly assigned participants (pregnant teenagers, aged 18 years and older who were at more than 24 weeks’ gestation) into three groups. The first group received a multicomponent home-based intervention, the second received a single-component home-based intervention, and the third received usual standard perinatal care. Results indicated that completion of two of more computer-assisted motivational intervention sessions, either alone or within a multicomponent home-based intervention, reduced the risk of rapid subsequent birth [32]. A review of theory-based interventions for contraception further supports MI as an intervention to prevent STIs [33].

These studies point to the potential effectiveness of an MI intervention to increase contraceptive use, reduce unintended pregnancies, and decrease STI incidence. We are the first to conduct a RCT using the MI intervention to alter contraceptive behaviors among incarcerated women, to assess its impact on contraceptive use other than condoms, and to separate pregnancy from STI prevention. Project CARE (Contraceptive Awareness and Reproductive Education) is designed to evaluate an innovative intervention, Motivational Interviewing with Computer Assistance (MICA), aimed at enhancing contraceptive initiation and maintenance among incarcerated women who do not want a pregnancy within the next year and who are anticipated to be released back to the community.

## Methods/Design

### Study objectives

The present study is designed to enhance our understanding of the efficacy of MICA to improve contraceptive use and safer sexual activity for incarcerated women.

The primary objectives are to: (1) increase the initiation of highly effective contraceptives while incarcerated; (2) increase the continuation of highly effective contraceptive use at 3, 6, 9, and 12 months; and decrease unsafe sexual activity.

### Participants

Participants will be recruited from the Rhode Island Department of Corrections (RI DOC) women’s division. All women between the ages of 18 and 35 years will be approached by a research assistant for study participation and after obtaining verbal consent will be screened for eligibility. This screening visit and all subsequent visits occur in a private location and study participation is not discussed with security staff.

Inclusion criteria include: (1) 18 to 35 years of age; (2) currently sexually active with men defined as having vaginal-penile intercourse at least monthly in the past three non-institutionalized months; (3) answered ‘No’ to ‘Do you plan to become pregnant within the next 12 months?’ (4) expected place of residence after release in Providence County or within 15 miles of follow-up site; (5) willing to comply with protocol, follow-up, and provide at least one locator; and (6) fluent in English.

Exclusion criteria include: (1) inability to give informed consent secondary to organic brain dysfunction, not having own legal guardianship, or active psychosis or otherwise unable to participate in the intervention or assessments (deaf, blind, or impaired communication skills that impair ability to participate in computerize assessment or counseling); (2) pregnant or trying to become pregnant within the next year; (3) hysterectomy, bilateral oophorectomy, tubal sterilization (by ligation or coils), intrauterine device (IUD), implantable contraceptive devices (Implanon) or other procedures which make it extremely unlikely to become pregnant; (4) women who are monogamous for more than 1 year with a partner who has had a vasectomy; (5) housed in segregation as we would be unable to recruit them for the study (once released from segregation a woman would then become eligible).

### Setting

This RCT was conducted at the RI DOC women’s facility, a state-run correctional system. It is a combined prison and jail housing both awaiting trial and sentenced individuals. It functions mostly as a jail with few women sentenced to more than 1 year. The average daily population is 240. Because many women are incarcerated more than once in a year the number of individual women who enter the RI DOC is less than 2,180, and the median length of stay is 3 days. The majority of women are White (51%), however African American (30.8%) and Hispanics (17.6%) are over-represented (state population 5.7% and 12.4%, respectively) [34]. Over 80% are under 40 years old.

### Procedures/Interventions

A research assistant (RA) will recruit potential participants in the housing units at the RI DOC. She will identify herself as non-RI DOC staff and inform women that study participation is completely voluntary and will not affect any privileges at the facility. Individuals who are interested in participating will be given an informed consent form to review and the study will be explained. Given that incarcerated populations are vulnerable populations with limited rights, the informed consent process is particularly important. The RA will detail that study participation is not linked to any extra medical or non-medical services in prison and will not influence parole status or any other jail privileges. Additionally, it will be explained that she does not need to participate in the study to receive reproductive health services. It will be explained that this is a confidential study but that if there is any information that a participant may harm herself, another person, or that a child or an elderly or disabled individual is in danger then that information will be reported. If there is a threat made to the security of the prison then prison staff will be informed. Counselors are instructed not to discuss study participation with any one at the RI DOC. All study staff are certified through the hospital in Human Subjects Protection and HIPAA, and a certificate of confidentiality has been obtained to further insure participant confidentiality.

Inclusion and exclusion criteria will be reviewed. If eligible and willing to participate in the study, the informed consent process will be completed (study explained, consent form read to the individual, and questions answered) and forms signed. Locator information will be obtained and consents signed for tracking [35].

Participants will be randomized by age (25 years or younger *vs*. older than 25 years), race/ethnicity (non-Hispanic White *vs*. other), and pregnancy intentions (wants no more pregnancies *vs*. wants a future pregnancy). Future pregnancy plans will be assessed by a single question: ‘Do you want to have any/anymore children?’ Based on these criteria there will be eight randomization groups. A random number generator will determine intervention status for the eight groups. Intervention status is then placed into sequentially numbered envelopes for the eight groups and each envelope is sealed and opened in order as women become eligible in each of the eight groups.

Before the first intervention session, a 45-60-minute computer-assisted questionnaire will be administered to the participant. From the baseline questionnaire, we will gather the following information: demographics, obstetric and gynecologic history, length of stay in prison, medical insurance, primary care provider or clinic, relationship violence, childhood abuse, addiction, and depression. These measures will aid in assessing the complex context in which a woman ‘chooses’ to initiate or not initiate contraceptive services.

The counselor/RA will then administer MICA or video according to randomization. Self-obtained vaginal samples for *Trichomoniasis vaginalis *, *C. trachomatis*, and *N. gonorrhoeae* will be obtained as well as a urine sample for a pregnancy test. Contraceptive, sexual, and drug-related risk behaviors will be measured via a calendar recall behavioral assessment, a Timeline Followback (TLFB) calendar of the prior 90 days at baseline and at each follow-up visit [36,37]. This assessment utilizes a calendar-based recall method using anchor dates to facilitate a more accurate response set. It also allows for a more thorough assessment of risk behaviors by allowing for the analysis of risk patterns by sexual partner, drug, and alcohol use and the co-occurrence of sexual- and drug-related risk, all within the context of life events. A detailed assessment of sexual behaviors and condom use with each of the participant’s other sex partners will be made by the participant.

#### *MICA*

Participants randomized to MICA will receive all core MI elements in the MICA sessions. A key component of any MI-based intervention is for the counselor to support autonomy by explicitly stating that it is the participant, not the counselor, who will make decisions regarding behavior change. Counselors will lead the participant in a directed, empathic discussion that includes a review of the Timeline Followback (TLFB) calendar, pregnancy intentions, the pregnancy and STI risk assessment printouts, and Stages of Readiness to Change. The behaviors targeted vary depending on the needs of the participant with the overall goal of decreasing the risk for unplanned pregnancies and STIs. For example, some want to focus on decreasing the number of sex partners, some using birth control consistently, and others want to see a medical provider to initiate a birth control method. The counselor will support the participant’s decision to make, or consider the possibility of, change. All MICA participants will receive: (1) feedback on personal risks for pregnancy and STIs; (2) clear advice to avoid pregnancy (until it is desired) and STIs that might impact future fertility by either using highly effective contraceptives and condoms consistently and correctly or staying abstinent; and (3) a review of the menu of options by which to prevent pregnancy and STIs. Detailed education about contraceptive choices is offered according to the participant’s needs and requests. The session will end with the development of a ‘CARE plan’ in which the participant lists her goals and methods for reaching her STI and pregnancy prevention goals. A second MICA session will be conducted 3 months after release and include a review of the’CARE plan’ as well as elements provided in session one. If a woman chooses no method or decides she wants to become pregnant then preconception counseling is offered.

#### *VIDEO*

Participants randomized to control videos will watch information on contraception and STI prevention that a participant would need to avoid exposure to intercourse that is poorly protected against pregnancy and against STIs. The baseline video is about contraception and is matched in time to the MICA sessions. At the 3-month post release session the video focuses on STIs, condom use, and preconception counseling.

At the end of the MICA or video session, the counselor will offer participants a list of community-based locations at which contraceptive services and STI screening can be accessed inexpensively or for free. Each participant will also be offered a referral to the Title X reproductive health educator at the jail. (In conjunction with RI DOC, the Title X program provides reproductive health services in jail and then transitional services in the community after release.) On-site contraceptive options include oral contraceptive pills, contraceptive patches, vaginal rings, and medroxyprogesterone injections. The counselor will suggest that the participant visit her primary care provider, the prison physician or one of the sites on the list for contraceptive supplies, annual pelvic examinations, and regular STI screening.

Follow-ups will occur at 3, 6, 9, and 12 months post release in the community or prison if re-incarcerated. This is a challenging population to track and retain in a longitudinal study. However, we have refined methods for tracking participants as they re-enter the community. In our previous trials, including Project WISE (PI Clarke R01DA024093), we have been able to locate and retain over 80% of participants for their follow-up assessments. Participants are asked to sign a letter for all locators that explains that they are participating in a research study and gives their permission to provide the study staff with information about the participant’s whereabouts if needed. Prior to each follow-up assessment, study staff attempt to contact participants to remind them of the appointment by letters and phone calls. The participant is asked to review and confirm or edit contact information at each visit. Financial compensation for the time and effort to provide assessment information and biologic samples also aids in study retention.

Questionnaire measures include contraceptive use, barriers to family planning services, relationship violence, substance use, depressive symptoms, pregnancy intentions and plans [20], sexual assertiveness, relationship status, and housing situation. In addition, participant biological outcomes will be assessed at each visit and include a self-obtained vaginal samples for *Trichomoniasis vaginalis C. trachomatis*, and *N. gonorrhoeae* as well as a urine sample for a pregnancy test.

### Primary hypotheses and outcomes

1. MICA will increase the initiation of highly effective contraceptives in jail more than educational videos. The primary outcome is initiation of a highly effective contraceptive method prior to release from jail. This will be measured by a review of the medical records and documentation of participants having received a contraceptive injection, pills, patch, ring, or other contraceptive device. Type of contraceptive and amount given will be recorded.

2. Women randomized to MICA will be more likely to continue the use of highly effective contraception at 3, 6, 9, and 12 months compared to women randomized to educational videos. The primary outcome is continued highly effective contraceptive use at 3, 6, 9, and 12 months. This will be measured through the TLFB calendars and confirmed with a review of the medical records. The secondary outcome is pregnancy as documented by a urine pregnancy test at each follow-up visit.

3. MICA will more effectively decrease unsafe sexual behavior more than educational videos. The primary outcome is an incident STI after a negative baseline test. The secondary outcome is intercourse that is poorly protected against STIs at 3, 6, 9, and 12 months as determined by the TLFB.

### Sample size

The sample size calculation is based on the biological outcomes and is the most conservative estimate. Data from our prior work (project CONNECT [20]) shows that the 6-month incidence of an STI was 33% [38]. We assume over a 12-month period this will increase to 43%. From this data, we assume that the intervention will decrease the incidence a moderate amount to 31%. Similarly, we assume a type I error of α = 0.05, an average within-subject correlation of 0.50 and a conservative estimate of a 20% attrition rate [39]. Using the similar procedure as above, the proposed enrollment of 400 participants (200 participants for each intervention) at baseline will achieve power of 80% to detect assumed differences after considering four repeated measures (3, 6, 9, and 12 months). Our proposed sample size may provide greater power to detect interaction effects, robustness to violations of model assumptions, protection against multiple comparison error rates, and increased efficiency to detect weaker relationships between variables.

### Statistical analyses

#### *Primary analyses*

Data analyses will be performed using the Statistical Analysis System, SAS 9.1.3 (SAS Institute, Inc, Cary, NC, USA). Following intention-to-treat principles, all participants who have been randomized to the two interventions will be included in the analyses. All significance tests will be two-tailed.

#### *Contraceptive initiation (Aim #1)*

Analyses of contraceptive initiation while incarcerated will include the percentage of participants starting a highly effective method requiring infrequent user maintenance (Subdermal implant, injectable contraception, or an IUD) as well as highly effective methods requiring more regular non-coital use (contraceptive pills, vaginal rings, and the patch). Initiation of contraception use (yes/no) will be analyzed using logistic regression to determine if women randomized to the MICA condition are more likely to initiate a contraceptive method while incarcerated than women randomized to the video condition. Baseline randomization characteristics (for example, age, race/ethnicity, and pregnancy intentions) will be incorporated in the initial model to insure randomization was successfully accomplished. Odds ratios and their 95% confidence intervals will be calculated for MICA using the video condition as the referent.

#### *Contraceptive continuation (Aim #2)*

Analyses of contraceptive continuation will include the assessment of highly effective contraception use (yes/no) at the four follow-up time points. In addition to assessment of contraception use, pregnancy intentions will also be assessed at each time point. Generalized linear mixed models (GLMM) will be used to analyze the binary outcome. GLMM allows for flexibility in the distribution of the outcome and can also incorporate repeated measures of both dependent and independent variables. Independent variables in the GLMM will include treatment group, pregnancy intentions, an indicator variable for time as well as risk factors for unplanned/unwanted pregnancy (education, health insurance status, alcohol/drug use, and pregnancy history). Testing the interaction between treatment group and time will inform us whether differences in contraceptive outcomes between treatment conditions become more or less pronounced over the 12-month follow-up.

#### *Incident STIs (Aim #3)*

Analyses of incident STIs will evaluate all STIs tested throughout the study. GLMM will be used, and we will follow the similar procedure outlined in Aim #2.

### Secondary analyses

#### *STI and pregnancy risk behaviors*

For this analysis we will utilize the TLFB data to identify the percentage of at-risk days that women engage in sexual activity not protected against an unplanned pregnancy. We will include use of all coital and non-coital contraceptive methods in this analysis, as well as sexual activity without a condom (not protected against STIs). GLMM will be used to test this hypothesis, and we will follow the similar procedure outlined in Aim #2.

#### *Predictors of contraceptive use*

Predictive variables will include demographics of age, race/ethnicity, and education as well as drug and alcohol use, depression, recent and childhood victimization, and pregnancy history. Following the stages of change, decisional balance, process of change, and self-efficacy will allow for an in-depth evaluation of the intervention. By following changes in these processes, the components of the intervention which most affect behavioral change can be determined.

Alternatively, we will also use measurement modeling to incorporate multiple measures, such as the self-reported primary outcomes and the incidence of pregnancy and STI in a latent factor that captures variance common to all measures [40]. Statistical analyses with latent variables allow us to minimize measurement error and thereby increase validity of the study.

#### *Treatment fidelity*

To promote treatment integrity, all treatment sessions will be audio-recorded and reviewed weekly by the treatment supervisor, who will use a MICA adherence checklist to assess session content and process as well as the MITI 3.1.1 scale to assess the quality of MI at each session [41,42]. Prior to enrolling participants, counselors will receive individualized feedback and coaching by a treatment supervisor who has reviewed every audio-recorded MICA sessions until the counselors are certified to competently conduct MICA counseling. Then counselors will receive feedback and coaching for the first 10 audio-recorded MICA sessions and over time will be provided with written feedback and coaching for every other to every third audio recordings. To insure fidelity an independent reviewer will code 10% of all coded MICA sessions using the Motivational Integrity Treatment Integrity scale. A counselor who falls below acceptable levels of proficiency in MICA will be identified and re-trained until able to demonstrate the necessary knowledge and skills.

## Discussion

The United States female jail population is a large population, particularly among women of childbearing age. Women leaving jail are at high risk for unplanned pregnancies and STIs and face many barriers to reproductive health services such as lack of insurance, transportation issues, and child care issues [43-47]. This research project is a RCT of an experimental intervention for incarcerated women at risk for unplanned pregnancies and STIs. It will compare changes in the initiation and maintenance of highly effective contraceptives and STI risk behaviors at multiple time points up to 12 months after release from the incarcerated setting. Improving contraceptive service utilization in this non-traditional setting has the potential to reach the women in the greatest need of services.

## Trial status

Recruitment for this trial is ongoing and expected to end by September 2012.

## Competing interests

The authors declared that they have no competing interests.

## Authors’ contributions

JGC conceived of the study, and participated in its design and coordination, and helped to draft the manuscript. MAG participated in the study design and helped to draft the manuscript. RES helped to draft the manuscript. MBR helped draft the manuscript. LARS participated in the study design and helped to draft the manuscript. All authors read and approved the final manuscript.

## References

[B1] Jones E Forrest J Henshaw SK, Silverman J, Torres A: Pregnancy, Contraception and Family Planning Services in Industrialized Countries 1989 New Haven, CT: Yale University Press

[B2] Henshaw SK Unintended pregnancy in the United States Fam Plann Perspect 1998 30 24 29 46 10.2307/2991522 9494812

[B3] Henshaw SK Women at risk of unintended pregnancy, 1990 estimates: the need for family planning services, each state and county 1993 New York, NY: The Alan Guttmacher Institute

[B4] Ventura SJ Mosher WD Curtin SC Abma JC Henshaw S Trends in pregnancies and pregnancy rates by outcome: Estimates for the United States, 1976–96 National Center for Health Statistics Vital Health Stat 2000 21 1 24 10635682

[B5] Sonfield A Kost K Gold RB Finer LB The public costs of births resulting from unintended pregnancies: national and state-level estimates Perspect Sex Reprod Health 2011 43 94 102 10.1363/4309411 21651708

[B6] Hellerstedt W Pirie P Lando HA Curry SJ McBride CM Grothaus LC Clark Nelson J Differences in preconceptional and prenatal behaviors in women with inteded and unintended pregnancies Am J Public Health 1998 88 663 666 10.2105/AJPH.88.4.663 9551015 PMC1508432

[B7] Korenman S Kaestner R Joyce T Consequences for infants of parental disagreement in pregnancy intention Perspect Sex Reprod Health 2002 34 198 205 10.2307/3097730 12214910

[B8] Kost K Landry DJ Darroch JE Predicting maternal behaviors during pregnancy: does intention status matter? Fam Plann Perspect 1998 30 79 88 10.2307/2991664 9561873

[B9] Han J Nava-Ocampo A Koren G Unintended pregnancies and exposure to potential human teratogens Birth Defects Res A Clin Mol Teratol 2005 73 245 258 10.1002/bdra.20132 15786494

[B10] Rosenberg KD Gelow JM Sandoval AP Pregnancy intendedness and the use of periconceptional folic acid Pediatrics 2003 111 1142 1145 12728127

[B11] Centers for Disease Control and Prevention Trends in reportable sexually transmitted diseases in the United States 2011 Atlanta, GA: CDC

[B12] Fleming DT Wasserheit JN From epidemiological synergy to public health policy and practice: the contribution of other sexually transmitted diseases to sexual transmission of HIV infection Sex Transm Infect 1999 75 3 17 10.1136/sti.75.1.3 10448335 PMC1758168

[B13] Trends in Sexualy Transmitted Diseases in the United States 2009 Data for Gonorrhea, Chlamydia and Syphillishttp://www.cdc.gov/std/stats09/trends2009.pdf

[B14] Forrest JD Epidemiology of unintended pregnancy and contraceptive use Am J Obstet Gynecol 1994 170 1485 1489 8178895 10.1016/s0002-9378(94)05008-8

[B15] Greenfeld LA Snell TL Women Offenders NCJ 1999 175688

[B16] Clarke J Rosengard C Rose J Hebert M Peipert J Stein M Initiation of birth control method among incarcerated women: comparison or pre-release and community delivery Am J Public Health 2006 96 840 845 10.2105/AJPH.2005.062869 16571698 PMC1470571

[B17] Centers for Disease Control and Prevention STDs in persons entering corrections facilities 2009 Atlanta, GA: CDC

[B18] Ruggiero L Transtheoretical model: applications in the prevention and treatment of cancer Med Pediatr Oncol 1998 Suppl 1 69 74 9659949 10.1002/(sici)1096-911x(1998)30:1+<69::aid-mpo10>3.0.co;2-w

[B19] Prochaska JO DiClemente CC Transtheoretical therapy: towards a more integrative model of change Psychother Theory Res Pract Train 1982 19 276 288

[B20] Miller WRRS Motivational Interviewing: Preparing People to Change Addictive Behavior 1991 New York, NY: Guilford Press

[B21] Butler C Rollnick S Cohen D Bachmann M Russell I Stott N Motivational consulting versus brief advice for smokers in general practice: a randomized trial Br J Gen Pract 1999 49 611 616

[B22] Saunders B Wilkinson C Phillips M The impact of a brief motivational intervention with opiate users attending a methadone programme Addiction 1995 90 415 424 10.1111/j.1360-0443.1995.tb03788.x 7735025

[B23] Swanson AJ Pantalon M Cohen KR Motivational interviewing and treatment adherence among psychiatric and dually-diagnosed patients J Nerv Ment Dis 1999 187 630 635 10.1097/00005053-199910000-00007 10535657

[B24] DE Smith HC Kratt PP Mason DA Motivational interviewing to improve adherence to a behavioral weight control program for older obese women with NIDDM: a pilot study Diabetes Care 1997 20 52 54 10.2337/diacare.20.1.52 9028693

[B25] Miller WR Benefield RG Tonigan JS Enhancing motivation for change in problem drinking: a controlled comparison of two therapist styles J Consult Clin Psychol 1993 61 455 461 8326047 10.1037//0022-006x.61.3.455

[B26] Group PMR Project MATCH secondary a priori hypotheses Addiction 1997 92 1671 1698 9581001

[B27] Miller WR Rollnick S Motivational inverviewing: Preparing people for change 2002 New York, NY: Guilford Press

[B28] Belcher L Kalichman S Topping M Smith S Emshoff J Norris F Nurss J A randomized trial of a brief HIV risk reduction counseling intervention for women J Consult Clin Psychol 1998 66 856 861 9803706 10.1037//0022-006x.66.5.856

[B29] Ingersoll KFL Sobell M Velasquez MM Project CHOICES Intervention Research Group. Reducing the risk of alcohol-exposed pregnancies: a study of a motivational intervention in community settings Pediatrics 2003 111 1131 1135 12728125

[B30] Ingersoll KS Ceperich S Nettleman MD Karanda K Brocksen S Johnson BA Reducing alcohol-exposed pregnancy risk in college women: initial outcomes of a clinical trial of a motivational intervention J Subst Abuse Treat 2005 29 73 80 10.1016/j.jsat.2005.06.003 PMC2875062 16183466

[B31] Peterson R Albright J Garrett J Curtis K Pregnancy and STD prevention counseling using an adaptation of motivationalinterviewing: a randomized controlled trial Perspect Sex Reprod Health 2007 39 21 28 10.1363/3902107 17355378

[B32] Barnet B liu J DeVoe M Duggan AK Gold MA Pecukonis E Motivational intervention to reduce rapid subsequent births to adolescent mothers: a community-based randomized trial Annals of family medicine 2009 7 436 45 10.1370/afm.1014 19752472 PMC2746510

[B33] Lopez L Tolley E Grimes D Chen-Mok C Theory-based interventions for contraception Cochrane Database Syst Rev 2009 1 CD007249 19160330 10.1002/14651858.CD007249.pub2

[B34] 2010 U.S. Censushttp://quickfacts.census.gov/qfd/states/44000.htm

[B35] Wei LJ Lachin JM Properties of the urn randomization in clinical trials Control Clin Trials 1988 9 345 364 10.1016/0197-2456(88)90048-7 3203525

[B36] Lea S Alcohol and drug use by abusers when incarcerated Addictive Behavior 1983 8 89 92 10.1016/0306-4603(83)90063-1 6880931

[B37] Sea M Comparison of two techniques to obtain retrospective reports of drinking behavior Addict Behav 1982 7 33 38 10.1016/0306-4603(82)90022-3 7080882

[B38] Willers DM Peipert JF Allsworth JE Stein MD Rose JS Clarke JG Prevalence and predictors of sexually transmitted infection among newly incarcerated females Sex Transm Dis 2008 35 68 72 10.1097/OLQ.0b013e318154bdb2 18090178

[B39] Clarke JG Anderson BJ Stein MD Hazardously drinking women leaving jail: time to first drink J Correct Health Care 2011 17 61 68 10.1177/1078345810385915 21278321 PMC4887099

[B40] Muther B Beyond S General latent variable modeling. Behaviormetrika 2002 29 81 117

[B41] Moyers T Martin T Manuel J Miller W Ernst D Revised Global Scales: Motivational Interviewing Treatment Integrity 3.1 (MITI 3.1) 2010 Alberquerque, NM: University of New Mexico; Center on Alcoholism, Substance Abuse and Addictions (CASAA)

[B42] Madson MB Campbell TC Measures of fidelity in motivational enhancement: a systematic review J Subst Abuse Treat 2006 31 67 73 10.1016/j.jsat.2006.03.010 16814012

[B43] Broyles RW McAuley WJ Baird-Holmes D The medically vulnerable: their health risks, health status, and use of physician care J Health Care Poor Underserved 1999 10 187 200 10.1353/hpu.2010.0498 10224825

[B44] Gispert M Brinich P Wheeler K Krieger L Predictors of repeat pregnancies among low-income adolescents Hosp Community Psychiatry 1984 35 719 723 6745880 10.1176/ps.35.7.719

[B45] Moos M Bangdiwala SI Meibohm AR Cefalo RC The impact of a preconceptional health promotion program on intendness of pregnancy Am J Perinatol 1996 13 103 108 10.1055/s-2007-994302 8672181

[B46] Radecki SE Bernstein GS An assessment of contraceptive need in the inner city Fam Plann Perspect 1990 22 122 127+144 10.2307/2135643 2379569

[B47] DeVille KA Kopelman LM Moral and social issues regarding pregnant women who use and abuse drugs Obstet Gynecol Clin North Am 1998 25 237 254 10.1016/S0889-8545(05)70367-3 9547769

